# Cultivated Sub-Populations of Soil Microbiomes Retain Early Flowering Plant Trait

**DOI:** 10.1007/s00248-016-0846-1

**Published:** 2016-09-21

**Authors:** Kevin Panke-Buisse, Stacey Lee, Jenny Kao-Kniffin

**Affiliations:** Horticulture Section, School of Integrative Plant Science, Cornell University, Ithaca, NY USA

**Keywords:** Arabidopsis, Cultivation-independent, Flowering, Microbiome, Selection

## Abstract

The collection of microorganisms found in the root zone of soil, termed the rhizosphere microbiome, has been shown to impact plant growth and development. Here, we tease apart the function of the cultivable portion of the microbiome from the whole microbiome in retaining plant traits modified through artificial selection on flowering time. Specifically, the whole microbiome associated with earlier flowering time of *Arabidopsis thaliana* was cultivated on four types of solid media to create cultivated fractions of the microbiome. These cultivated microbiomes were subsequently preserved in glycerol, frozen, and revived to yield a portion of the cultivable fraction to compare (1) whole microbiome, (2) cultivable microbiome, and (3) revived, cultivable microbiome controls on early flowering time. Plants grown in soils inoculated with bacteria grown on 25 % Luria broth and 10 % tryptic soy agar retained the early flowering trait. An increase in leaf biomass with two of the cultivated microbiomes (49.4 and 38.5 %) contrasted the lowered biomass effect of the whole microbiome. Inoculation with the cultivated microbiomes that were cryopreserved in glycerol showed no effect on flowering time or leaf biomass. The results indicate that the cultivable portion of a plant’s microbiome retains the early flowering effect in *A. thaliana*, but cryopreservation of the cultivated microbiomes disrupts the microbial effects on flowering time. Furthermore, the contrasting effects on leaf biomass (an indirect response from selection on early flowering time), seen with the whole microbiome versus the cultivable portion, suggests versatility in using cultivation methods to modify multiple traits of plants.

**Electronic supplementary material:**

The online version of this article (doi:10.1007/s00248-016-0846-1) contains supplementary material, which is available to authorized users.

## Introduction

Several studies have emerged exploring the potential of plant-associated microbiomes to influence a wide range of traits, including growth, disease suppression, stress tolerance, and flowering [1–4]. Traditionally, most investigations of host-microbe interactions tend to look for and isolate one or a few strains responsible for a change in host phenotype. While isolates are convenient for studying host-microbe interactions, recent work suggests that complex microbial communities, from consortia of just a few to whole microbiomes, may show more robustness in altering complex host traits [4–7]. Increasingly, more studies are examining the whole microbiome effects on plant traits [7–9]. This line of inquiry has led to greater interest in complex host-microbe and microbe-microbe interactions [5, 10, 11]. Whole microbiome investigations are not without their constraints, however. The complexity of whole microbiomes makes identification of the actual players driving the host responses difficult to decipher. Cultivation of whole microbiomes, without isolation of single strains, is presented here as a method to reduce the complexity of the microbial communities while retaining the key microbial players and preserving their collective functions.

The ability to assemble functional microbiomes is an important step to expanding both fundamental and applied research involving plant-microbiome interactions. Microbiome assembly is primarily approached from the bottom-up, relying on the addition of candidate taxa by either phylogenetic or functional genotypic/phenotypic criteria [12]. Alternatively, we used directed selection in a previous study to assemble plant microbiomes associated with flowering time of multiple plant hosts and found distinct bacterial community profiles assembling by flowering time [7]. This represented a departure from existing methods by applying a top-down approach that replaces strain or species function with whole microbiome function as a selection criterion. Interestingly, microbiomes also exhibited effects on secondary traits (indirect selection), including differences in biomass. The novelty of the study was the ability to generate microbiomes using a cultivation-independent approach and transferring these microbiomes into the soils of new hosts to reproduce the selected plant trait. However, the role and interactions of the key microbial players remained elusive even with robust sequencing data (12,000 reads per sample). This was due, in part, to the fact that shifts in flowering time corresponded more to patterns of presence/absence of bacterial taxa than patterns of relative abundance. This emphasis occurred because many taxa appeared in all of the samples, indicating a shared, core microbiome that likely had little to no effect on flowering time.

In this study, we examined an early flowering microbiome enriched over 16 generations of selection in comparison to cultivated and cryopreserved subsets of the early flowering microbiome for their ability to reproduce effects on plant traits in *Arabidopsis thaliana*. Cultivated subsets were derived from soil and water mixtures incubated on four solid media. Revived microbiomes were derived from cultivated microbiomes preserved in glycerol at −80 °C. Flowering was considered the primary trait modulated by the microbiome and cultivated microbiomes, while other attributes such as soil pH and plant biomass were considered secondary traits. Our objective is to compare the primary and secondary trait effects of whole microbiomes, cultivated microbiomes, and revived microbiomes to demonstrate the potential of using sub-populations of microbiomes to modify plant traits. We hypothesized that inoculation of the early flowering cultivable subsets of the microbiome into *A. thaliana* soils would reproduce the flowering response, but that variation in microbiota across the different growing media and cultivation methods would lead to variations in the secondary plant trait (biomass) indirectly selected on through the multi-generation study.

## Methods

### Growth Chamber Conditions

Plants were grown at a constant 22 °C under a 16-h/8-h day/night cycle in a growth chamber (Percival-Cornell University Weill Hall Life Sciences Growth Chamber Facility, Ithaca, NY, USA). Light intensity at plant height was 250 UE m^−2^ s^−1^. Relative humidity was set to 70 % for the duration of the study.

### Plant Growth Conditions

Seeds across all phases of this study came from a static, non-changing seed pool of a highly inbred line, *A. thaliana* Col-0 (Lehle Seeds Co., Round Rock, TX). Seeds were used from this common seed pool to fix allelic frequencies across all phases of this study and to ensure that any changes in plant traits when compared to controls or other phases of the study are the result of microbiome inoculation. All microcosms were watered from bottom reservoirs.

### Assembly of Early Flowering Microbiomes

Inoculants for early flowering microbiomes were generated through an iterative selection process detailed previously [7, 13]. Microcosms (*n* = 14, 7.6 cm diam. pots) of ~100 *A. thaliana* individuals grown in a 1:1 mixture of field soil/potting mix soil (Lambert General Purpose Mix) were prepared. *A. thaliana* was chosen as the model plant for this process because of its fast generation time and well-characterized growth requirements and physiology. The field soil was obtained from a collection of sites across Ithaca, NY, USA (42.456583, −76.368822; 42.452265, −76.369477; and 42.414913, −76.442272) representing agricultural, forest, and grassland soils. The mixed environment soil was added to provide a diversity of soil microorganisms for the initial generation. The potting mix was autoclaved prior to each generation, and after the first generation, it became the sole growing medium for the remainder of the study. In each generation, a subset of soil from the four pots displaying the earliest flowering was set aside as the inoculant for the next generation. Biomass and soils were harvested immediately following flower bolting of >90 % of the individuals within all pots. Control pots consisted of the plants and steam-sterilized soils, but the units were not inoculated with early or late-flowering microbiomes.

**Fig. 1:**
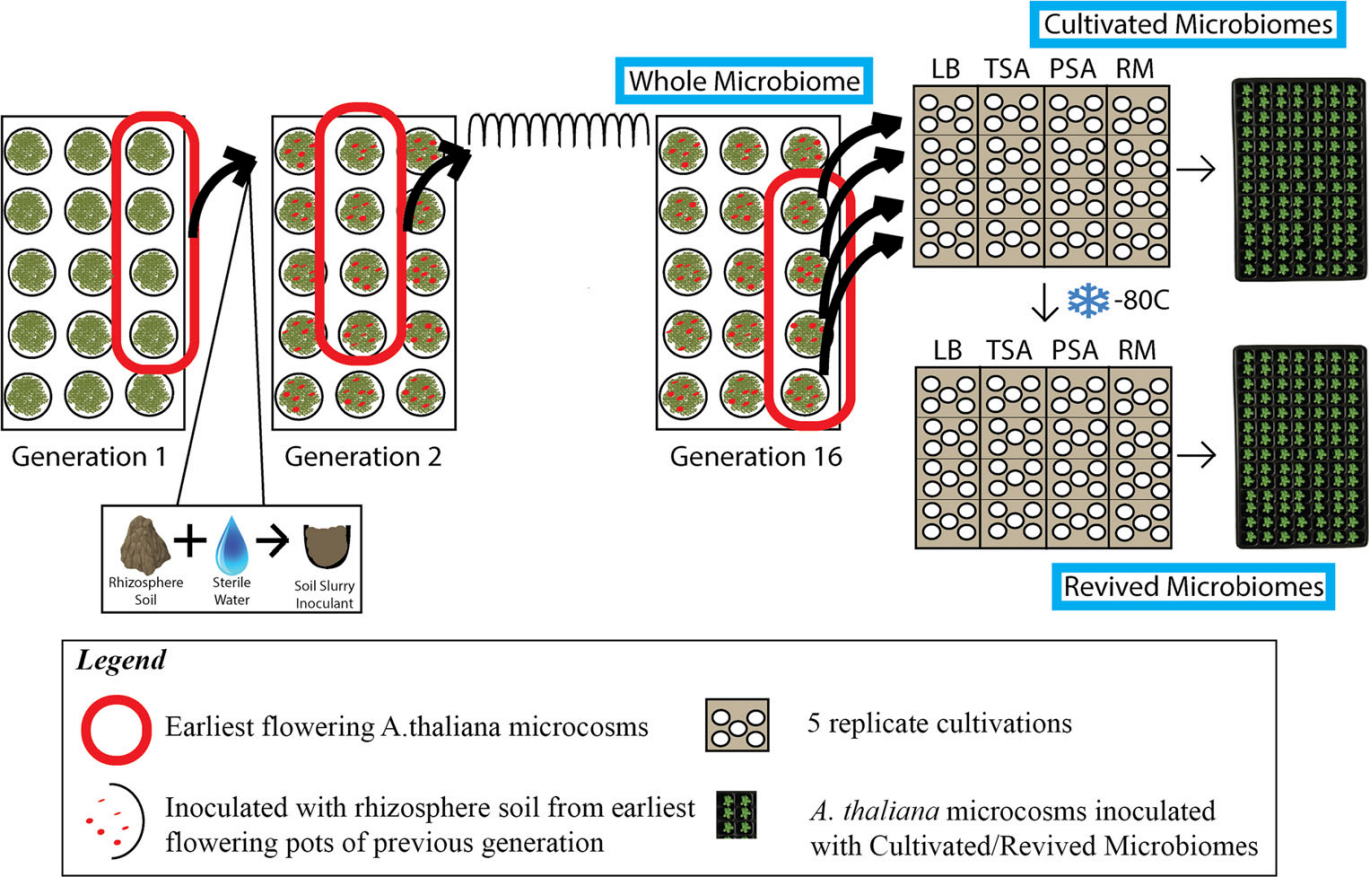
Experimental design diagram. Diagram of the experimental design, beginning with the iterative selection of *A. thaliana* rhizosphere microbiomes for earlier plant flowering, and including the three experiment phases (outlined in *blue*) detailed in this paper. The three phases are (1) whole early flowering microbiome, (2) cultivated microbiomes derived from the whole early flowering microbiome, and (3) the cryopreserved, revived microbiomes derived from the cultivated microbiomes. The whole microbiome is the 16th generation of microbiome selection for induction of earlier flowering in *A. thaliana*. The rhizosphere soil of this generation from the four earliest flowering microcosms was blended into a slurry and used to inoculate five replicate plates of four solid media. Streaks from the resulting microbial lawns (cultivated microbiomes) were used as inoculants in *A. thaliana* microcosms to assess effects on plant growth and development in comparison with the whole microbiome and controls. Another streak of the cultivated microbiome plates was used to create glycerol stocks that were frozen at −80 °C. These cryopreserved cultivated microbiomes were then revived and cultivated again on their respective solid media and then used as inoculants in *A. thaliana* microcosms. All *A. thaliana* seeds used in the experiment are from a homogenized stock of Col-0 seed, and no seed was saved or transferred between generations or phases. (Color figure online)

In this study, the subsets of soil for inoculation were pooled and prepared by combining 180 mL of sterile, deionized water and 35 g of the harvested rhizosphere soil. The mixtures were shaken vigorously for 60 s upon preparation and periodically during transfer. The autoclaved soil in each pot of the subsequent generation was inoculated with 12 mL of the mixture. The control group pots were treated with a sterile inoculant. Plants were watered with a 10 % solution (10 ppm N, 10.5 % nitrate/89.5 % urea) of 20-10-20 Jack’s Professional General Purpose Fertilizer (J.R. Peters, Inc., Allentown, PA, USA). The low level of available nutrients in the potting medium as well as in the watering regime ensured that the plants were under nutrient limitation, providing a strong filter to impose microbiome effects on soil nutrient mineralization. As the genetic pool of the plants was held constant using a highly in-bred genotype, the only adaptive traits to advance over generations were limited to the trait-associated microbiota derived from the soil inoculation. This selection process continued for 15 successive plantings to develop distinct, trait-associated soil microbiomes associated with early flowering time. A visual depiction of this process and the overall experimental design is diagramed in Fig. 1.

### Cultivation

Cultivation methods were employed to test the ability of the cultivable fraction to reproduce the function of the early flowering microbiome. Inoculant slurries for cultivation were prepared by combining 30 g of trait-associated rhizosphere soil from each of the four pots that displayed earliest flowering in the 16th iteration of the selection process and 25 mL of sterile, deionized water in a 50-mL tube and shaking the mixture for 1 min. Soil was pelleted at 3500×*g* for 30 min, and 750 μL of supernatant was inoculated onto each of five replicate plates and spread using a flame-sterilized glass spreader. The plates were incubated at 25 °C in the dark for 7 days. Glycerol stocks (25 %) of all plates were made from a streak and stored at −80 °C for the revival portion of the study.

The four solid media (25 % Luria broth (LB), 10 % tryptic soy agar (TSA), pseudomonad selective agar (PSA) [14], and rhizosphere medium (RM)) were prepared according to the recipes in Table S1. The “rhizosphere medium” was prepared by blending *A. thaliana* rhizosphere soil with agar (50 % soil by volume) and autoclaving. Selective agents (antibiotics and cycloheximide) were filter sterilized and added after autoclaving media, immediately prior to pouring.

### Reproduction of Function in Cultivated Fractions of Early Flowering Microbiomes (from Fresh Soil)

The effects on plant growth of the four cultivated microbiome fractions were assessed by inoculating each into the *A. thaliana* microcosms. A 1 × 3-cm streak was collected from each plate with a glass scraper and suspended in 2 mL of phosphate-buffered saline (PBS). This sub-sampling method ensures the collection of multiple colonies of bacteria, which allowed us to test for the effects of mixed, cultivated sub-populations of the microbiome, instead of single isolates. Care was taken to avoid carryover of any solid media. Sixty microliters of these suspensions was then inoculated into a plug flat containing steam-autoclaved Lambert General Purpose Mix. The surfaces of the plug flats were sprayed with sterile water to saturate the potting soil. After 48 h, five *A. thaliana* Col seeds were sown into each plug. Sterile water-inoculated plugs served as controls. Sixty microliters of PBS was added to half of the control plugs to control for any effect of the PBS in the inoculants on plant growth. The different treatment and control plugs were situated within the flats at random. Domes were added to create high-humidity conditions until germination and establishment, after which they were removed. Flowering times were recorded following the complete flower bolting of a microcosm. Both leaf and reproductive tissue biomass were harvested and dried at 50 °C until constant weight.

### Reproduction of Function in Revived Microbiomes

The effects on plant growth of the cryopreserved and subsequently revived, cultivated microbiome fractions were also assessed in *A. thaliana* microcosms. The frozen glycerol stocks of bacteria were revived for both liquid and solid cultivation. For the liquid cultivation method, glycerol stocks of bacteria were inoculated into 1 mL of the respective medium in which they were originally cultured, but without selective agents (antibiotic and antifungal) or agar. These were then incubated for 4 h at 25 °C. Starter cultivations of 250 μL were then transferred into 5-mL liquid cultures containing the selective agents detailed in Table S1. For the plate method, inoculant was retrieved from the glycerol stock and placed into 200 mL of the respective medium, incubated for 1 h, and plated onto the respective solid medium, complete with selective agents. Two replicates were prepared for each glycerol stock sample (solid and liquid), and all cultivations were incubated at 25 °C in the dark.

Cultivated microbiomes were incubated for 5 days and were inoculated randomly into plug flats of steam-autoclaved Lambert General Purpose Mix. Growing conditions and sample collection were as described in the section. For the plate method, a streak of the plate colonies was suspended in PBS. Then, 60 μL of either liquid cultivation or a PBS slurry of the solid medium cultivation was inoculated into each plug. Duplicates of each replicate were inoculated to mitigate error from edge effects and microclimatic variation. Sterile water and PBS control plugs identical to those described in the previous section were also randomly placed.

### 16S rRNA Gene Sequencing

DNA was extracted from frozen soil samples using the PowerSoil DNA Isolation Kit (MO BIO Laboratories, Inc., Carlsbad, CA, USA) according to the recommended protocol for highly organic soil. Approximately 0.15 g of soil from each sample was used for isolation of DNA. Quantification was performed with the standard dsDNA quantification protocol for PicoGreen (Thermo Fisher Scientific, Inc., Waltham, MA, USA). All pipetting for DNA extraction was conducted with an Eppendorf epMotion 5075 pipetting robot (Eppendorf AG, Hamburg, Germany). We amplified 16 S ribosomal RNA (rRNA) gene sequences in duplicate from the extracted DNA. The PCR primers used are those described in Caporaso et al. (2012) that target the bacterial/archaeal 16S rRNA gene variable region 4 (515 F/806 R) for downstream paired-end Illumina (Illumina, Inc., San Diego, CA, USA) barcoded sequencing [15]. Amplicons were quantified with PicoGreen, and 200 ng of each sample was pooled and purified with the desalting protocol of the Qiagen QIAquick Spin Filter Purification Kit (Qiagen Inc., Valencia, CA, USA). The amplicon pool was submitted to the Cornell Life Sciences Sequencing Core with the sequencing primers detailed in Caporaso et al. (2012).

### Statistics and Sequence Analysis

Plant trait data were analyzed by analysis of variance (ANOVA) using JMP 10 (SAS Inc.). Significance between groups was determined at alpha level 0.05. Contrasts between means were found using post hoc analysis (Fisher LSD and Tukey’s HSD).

For 16S rRNA gene sequence analysis, paired-end reads were truncated at the first low-quality base and quality filtered to remove those with an average quality score below 25, fewer than 200 bases, ambiguous bases, primer mismatches, erroneous barcodes, and homopolymer runs exceeding six bases. Paired-end reads were merged with ea-utils [16] and then demultiplexed within the QIIME software package (Qiime.org) [17]. Operational taxonomic units (OTUs) were picked against the Greengenes Database [18] with uclust [19]. Sequences with identity below 60 % and plant chloroplast or mitochondrial 16S rRNA sequences were filtered from the dataset. The smallest number of sequences belonging to any sample was 9799. This value was used to rarify all samples to that number of input sequences for analysis requiring even sample sizes for robust results. Alpha diversity measures (within-sample diversity) were calculated with Strong’s dominance [20]. Beta diversity measures (between-sample diversity) were computed with weighted UniFrac, and the resulting distance matrix was used to create the principal coordinates plot [21]. The heatmap of key taxa was created from the log relative abundance of all taxonomic Orders that differ significantly between samples exhibiting early flowering/biomass shifts and those that did not. These were then classified by the Prediction Analysis for Microarrays for the R package, which uses the least shrunken centroid method [22]. Log2 fold change and significance of taxa shifts were computed using the DESeq2 method [23]. The inputs used for DESeq2 were only taxa present in >80 % of the samples of a given phenological group (e.g., early flowering vs. no flowering effect); in other words, the core microbiomes associated with the plant phenotype effects.

## Results

### Whole Microbiome Effect on Flowering Time

**Fig. 2:**
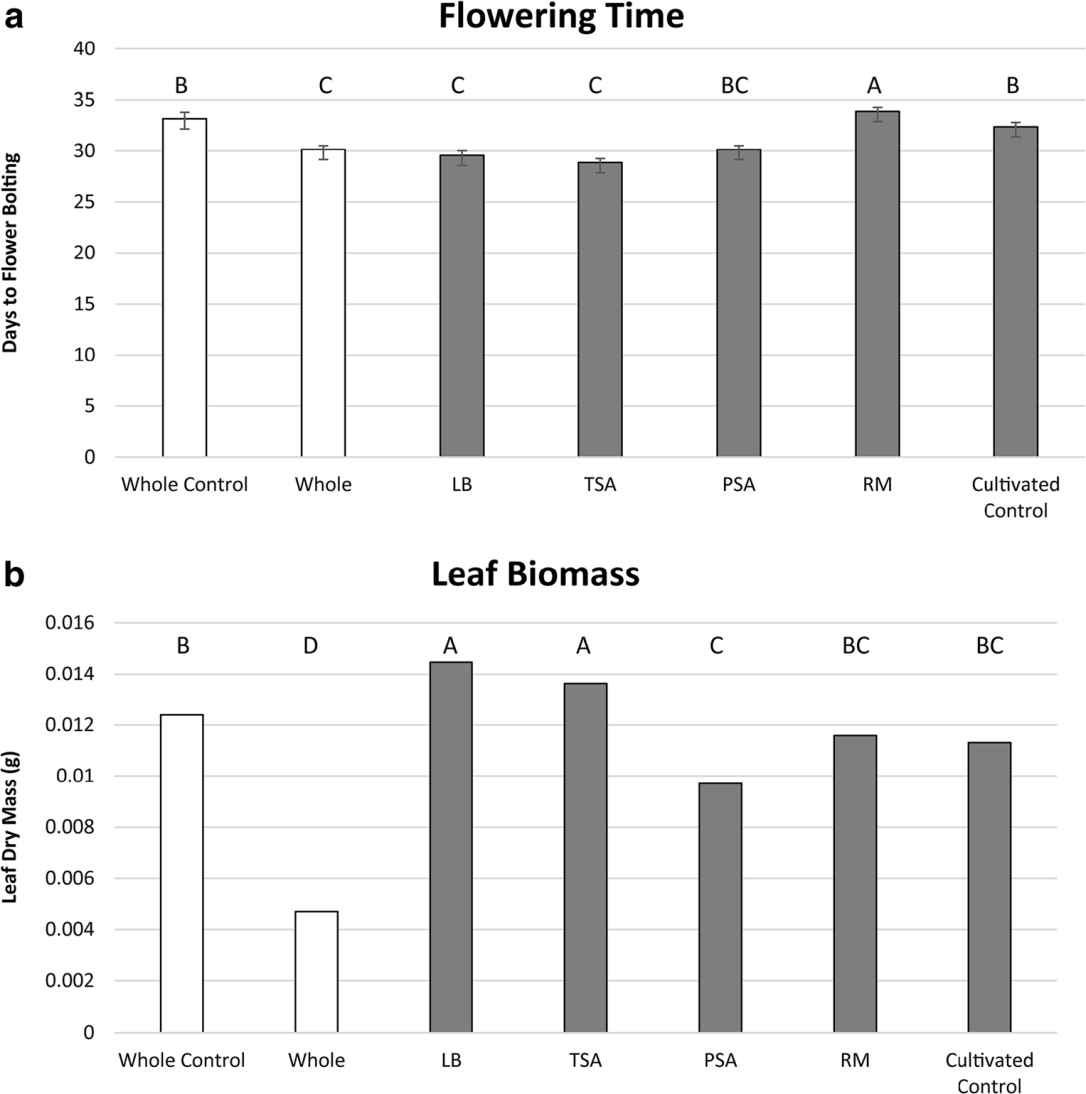
Inoculant effects on flowering time and leaf biomass. Comparison of whole microbiome and cultivated microbiomes’ effects on **a** flowering time (days to flower bolting of >90 % of individuals) and **b** leaf biomass (dry weight). The whole and cultivated microbiome phases each have distinct controls in which plants were inoculated with sterile soil (whole microbiome) or sterile water (cultivated microbiome). Whole microbiome phase bars are *unshaded* and cultivated microbiome phase bars are *shaded gray*. Media abbreviations: *LB* 25 % Luria broth; *TSA* 10 % tryptic soy; *PSA*: Pseudomonad semi-selective; *RM* rhizosphere

Inoculation of whole early flowering microbiomes into soils of *A. thaliana* genotype Col led to decreases in leaf biomass and fewer days to flowering, when compared to the control. Reproductive tissue biomass was unaffected by treatment in all three phases of the experiment. Similarly, flowering time decreased in the treatment group by 9.1 % compared to the control group: 30.15 ± 0.38 and 33.17 ± 0.63 days, respectively (Fig. 2a). Individual plant leaf biomass in the treatment group decreased by 61.2 % from the control group: 0.0047 ± 0.0014 and 0.0124 ± 0.0022, respectively (Fig. 2b).

### Cultivable Microbiome (Cultured from Soil) Effect on Flowering Time

Cultivated microbiomes showed significant differences in both leaf biomass and days to flowering from control microcosms and one another. PBS was used as an isotonic solution to suspend cultivated inoculants prior to inoculation. The PBS-inoculated controls and sterile inoculant controls were compared to determine if the addition of PBS altered plant growth. PBS showed no effect on plant growth (Table S2). Flowering responses in the culturing phase were also significant: 8.7 and 10.9 % earlier than the control for LB and TSA media, respectively, and 4.7 % percent later for RM (Fig. 2a). Leaf biomass was characterized by significant increases of 49.4 and 38.5 % for LB and TSA media, respectively (Fig. 2b).

### Revived Microbiome (from Cryopreserved Cultivated Microbiomes) Effect on Flowering Time

Revived microbiome inoculation yielded no significant differences in plant biomass from control microcosms (Table S2). In addition, no flowering effect was observed for any of the treatment groups, indicating a complete loss of treatment effect (Table S2). PBS had no effect on plant growth (Table S2).

### Control Comparisons

Phases were analyzed independently of one another due to the difference in microcosm size between the whole microbiome phase and the culturing and revival phases. In order to ensure the robustness of comparing results between phases, we also compared control groups across all phases. There was no significant difference between control groups across phases for either flowering time or leaf biomass.

### 16S rRNA Gene Sequencing Analysis

**Fig. 3:**
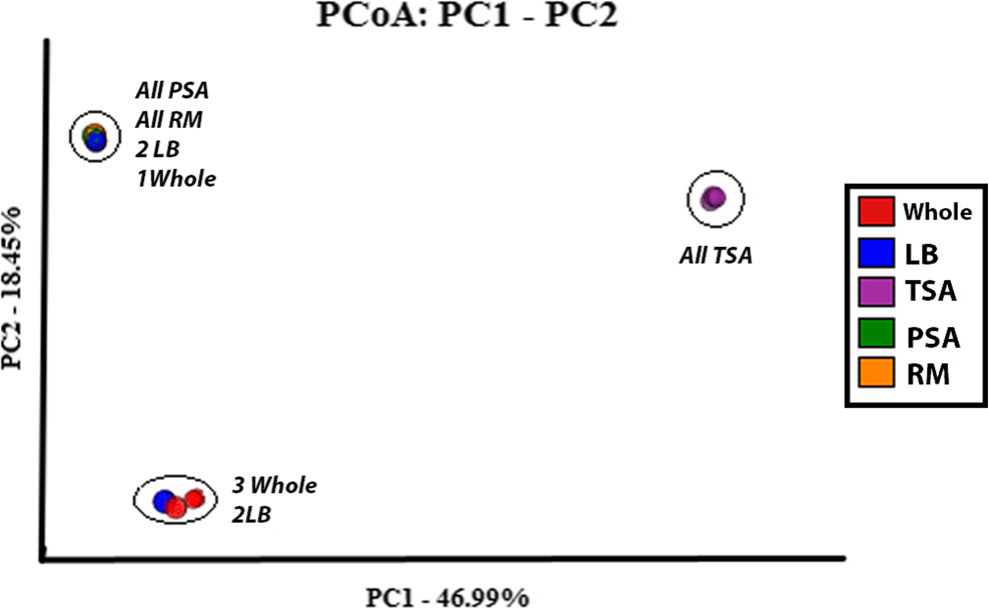
Weighted UniFrac principal coordinates plot. Principal coordinates plot of weighted UniFrac distance matrix illustrates the similarities and differences within and between sample groups. Weighted UniFrac distances show separation of the microbiome treatments by microbial community composition. Weighted UniFrac distances are sensitive to relative abundance of observed OTUs and reveal patterns and differences in the abundance of taxa. Samples were rarefied to an even sampling depth of 9799 seqs per sample based on the sample with the smallest number of sequences. Percentages on each axis represent the percent variation explained by each of the PCs. Close proximity of points obscures individual colors. *Circles* have been added around clusters, and sample points within each cluster are listed adjacent to each cluster

**Fig. 4:**
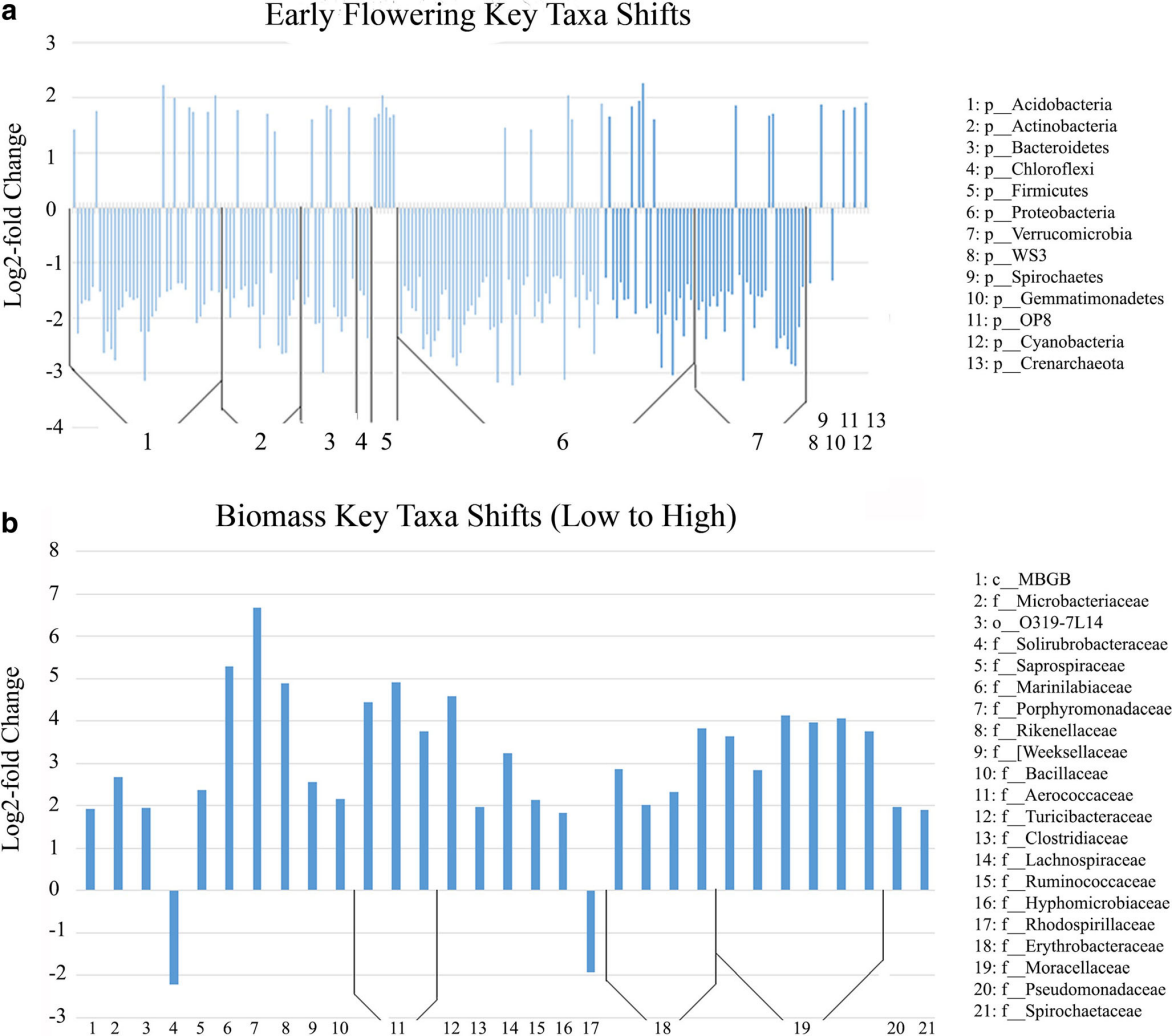
**a**, **b** Log2 fold change in abundance of taxa associated with shifts in flowering time and biomass. Key taxa were identified by analysis with DESeq2 differential abundance analysis. **a** Only taxa present in >80 % of the samples that showed an early flowering effect and those present in >80 % that showed no effect on flowering were used as inputs for DESeq2 in order to assess the core trait microbiome. Relativized log (log2 fold change) bars are grouped by phylum to assist in delineations between taxa groups. **b** Only taxa present in >80 % of the samples that showed a low biomass effect and those present in >80 % that showed a high biomass effect were used as inputs for DESeq in order to assess the core microbiome. Relativized log (log2 fold change) bars are grouped by closest shared taxonomic level to assist in delineations between taxa groups. Taxa preceded by “c__” are classes, “o__” are orders, and “f__” are families

**Fig. 5:**
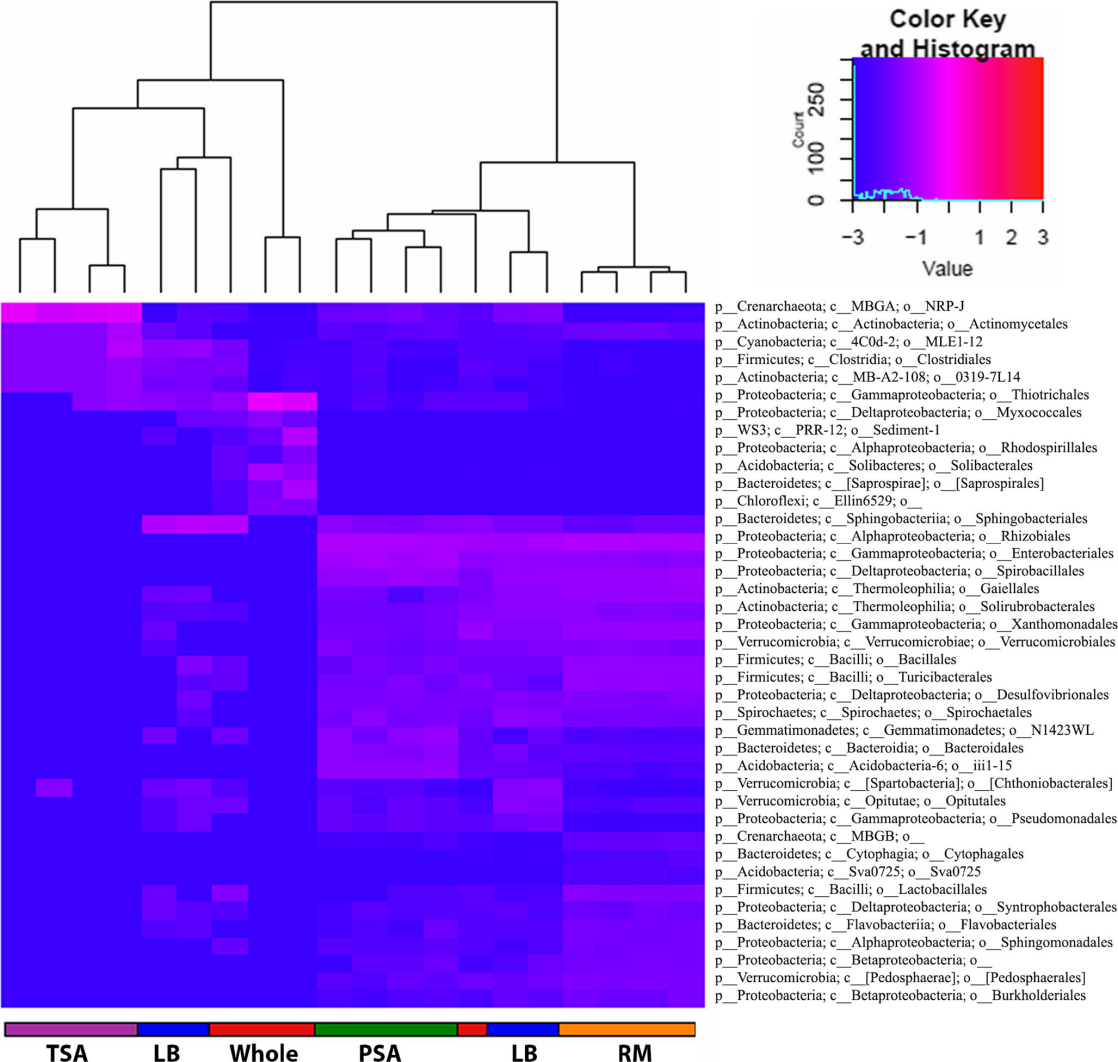
PAMR heatmap of key taxa. Heatmap of log relative abundance of key OTUs associated with observed phenotype effects identified by DESeq2. *Columns* represent individual samples and cluster primarily by treatment group. The *rows* represent OTUs at the order level. *Dendrograms* on each *axis* illustrate the relationship between the *columns* and *rows*. The *color key* at the *top left* includes a frequency histogram of number of OTUs at each log expression level. OTUs with zero expression were changed to 0.001 to allow the use of a log transformation. The whole microbiome and LB groups are the only two groups from which samples do not cluster correctly. Two LB samples and one whole microbiome sample do not group with their corresponding treatments

Bacterial community patterns showed visible shifts in taxa abundance between the whole and cultivated groups (Fig. S1). The whole microbiome and LB-cultivated groups showed significant variation within groups as well. Principle coordinates analysis (PCoA) illustrates the within-group variance in the whole microbiome and LB groups; the uniformity of the TSA, PSA, and RM groups; and the relationship between all of the treatment groups (Fig. 3). The results of the DESeq2 analysis produced a list of only 197 OTUs that differ significantly between samples exhibiting an early flowering effect and those that did not (Fig. 4a). In addition, the shift from low biomass to high biomass was characterized by significant shifts in only 31 OTUs (Fig. 4b). These 228 “key taxa” were used as the input for the PAMR heatmap to visualize the shifts between groups (Fig. 5).

## Discussion

We show that cultivation of mixed strains or taxa from rhizosphere soil presents potential benefits over the use of single isolate methods to modify a plant trait. However, approaches to preserve and revive these cultivable microbiomes resulted in a loss-of-the-trait effect. Specifically, the inability to maintain the flowering and biomass effects through cryopreservation and revival of the cultivated microbiome is likely a function of poor survival of taxa associated with these plant traits and selection for taxa that are tolerant of cryopreservation [24, 25]. The cultivable microbiome, while less complex than the whole microbiome, appears to retain a sufficient portion of the microbial community responsible for the early flowering effect. The loss of this effect in the revived cultivable microbiome indicates the need to study the role of complex communities in plant-microbiome interactions. In addition, inoculation of the cultivated sub-populations into the soils containing *A. thaliana* led to unexpected changes in plant flowering and leaf biomass responses.

Previous speculation on the driving processes behind microbiome-mediated shifts in flowering time include alteration of environmental cues tied to flowering (photoperiod and vernalization), pathogen pressures, and nutrient availability stresses [4, 7, 26]. Results of this study, however, suggest that the factors driving flowering time modulation may not be so straightforward. The whole microbiome treatment was characterized by significant decreases in flowering time and leaf biomass, which is consistent with low-nutrient or non-lethal pathogen accumulation stress responses [27]. However, two of the cultivated microbiomes (grown on LB and TSA) retained roughly equivalent decreases in flowering time, but exhibited ~40–50 % increases in leaf biomass in comparison to controls. These observations suggest that the plant-microbe-environment interactions that induce the primary early flowering response are not necessarily linked to the secondary leaf biomass response. The increase in biomass observed in the cultivated microbiome phase is consistent with previous studies on initiation of flowering in *A. thaliana* [28, 29]. This may indicate that cultivation of the early flowering microbiome can eliminate deleterious members of the microbiome responsible for the decrease in leaf biomass in the whole microbiome. Furthermore, decoupling of the early flowering and low-biomass traits could potentially suggest a more direct role of the microbiome in flowering time modulation.

The differences in biomass and flowering responses between cultivation media present cultivation as a strategy for eliminating undesirable taxa from microbiomes. Cultivation and inoculation into a sterilized rhizosphere can disrupt existing associations between microorganisms of the whole microbiome and potentially change interactions with the new plant host. Similar disruption of established plant-microbe associations has been observed to change overall community function [30]. In this regard, choice of cultivation medium and plant host appear to play a crucial role in determining microbial succession dynamics within the new host rhizosphere and, in turn, overall function in the plant host. This is supported by the observation of larger, more dominant community members in the whole microbiome treatment and smaller, more numerous members in the cultivated treatments, as determined by alpha diversity indices [20].

Community analysis revealed that the taxa shifts apparently driving these effects come down to a very small percentage of the overall community. The early flowering effect is characterized by shifts in only 197 key taxa, including overall decreases in abundance of certain Actinobacteria, Acidobacteria, Bacteroidetes, Proteobacteria, and Verrucomicrobia and increases in Spirochaetes, Firmicutes, and the Archaea Crenarchaeota (Class MBGA) (Fig. 4a). The biomass effect was characterized by shifts in just 31 key taxa between the low and high biomass effects (Fig. 4b). The high biomass effect was represented by relative increases in select Firmicutes, Bacteroidetes, Spirochaetes, Proteobacteria, and the Archaea Crenarchaeota (Class MBGB) and relative decreases in Actinobacteria. Only 6 of these 228 taxa are associated with both effects. Furthermore, many of these taxa are virtually unstudied and lie outside the traditional plant growth-promoting groups that typically include Pseudomonads, Rhizobia, Azospirillum, Bacillus, Streptomycetes, Azotobacter, and Agrobacterium [31].

Taken together, these results highlight cultivation as a method for simplifying microbiome communities while retaining, enhancing, or modifying microbiome function. A lack of mechanistic understanding currently limits studies of bioinoculant efficacy for commercial production [32]. The associated reduction in complexity and changes in cultivated microbiome effects between cultivation media supports the utility of using sub-populations of the microbiome in pursuing mechanism-level understanding of plant-microbe interactions. Cultivation may also contribute to the development of novel technologies and processes for plant production systems. Current methods of commercial bioinoculation can be readily adapted for use with more complex cultivated microbiomes over single-strain inoculants [33]. The biomass differences observed between plants grown with the whole and cultivated microbiomes illustrate the potential of cultivation for maintaining primary plant traits of a microbiome and modulating secondary traits. For example, a microbiome that accelerates time to flowering and increases plant biomass is well-suited for agriculture and could reduce production time and costs. Finally, based on our microbial community profile data, cultivation and subsequent reintroduction to the rhizosphere appears to allow for the transfer and enrichment of taxa that cannot be isolated in culture.

We have presented the cultivation of microbiomes here as a potential tool to narrow down microbiome components that influence specific plant phenotypes. Specifically, it allows for functional screening of a segment of the microbiome previously identified as having a link to a plant trait. More detailed screening of specific bacterial strains or mixtures of strains on the development of a plant phenotype would facilitate more mechanistic-level understanding of how bacterial communities interact with plants. We showed that the ability of cultivated microbiomes to both reproduce the primary plant response (flowering time) and modulate the secondary response (leaf biomass) contributes strongly to evidence in support of microbiome use in plant production systems.

## Electronic supplementary material

Below is the link to the electronic supplementary material.

ESM 1 (DOCX 676 kb)
