# Supplementary material for: Cultivated Sub-Populations of Soil Microbiomes Retain Early Flowering Plant Trait
Source: Microb Ecol. 2016 Sep 21;73(2):394–403. doi: 10.1007/s00248-016-0846-1 (PMC5272889; doi:10.1007/s00248-016-0846-1)

**Supplement**

Supplementary Table and Figure Legends

Table S1. Recipes for Solid Media

The preparation protocols for the four solid media used in this study.

Table S2. Non-significant supplemental data. A) Cross-study control validation comparing the control flowering time and biomass values across study phases and designs to support the robustness of comparisons within study phases. B) Flowering time and plant biomass values of phosphate-buffered saline controls in the cultivation and revival phases. C) Flowering time and plant biomass values for the revival phase. All + values are standard error and none of the values presented are significantly different at alpha-level 0.05.

Figure S1. Histogram of Relative Abundances Summarized by Group

Bar chart of relative abundance of phyla summarized within groups. Each color represents a phylum and its size corresponds to the relative abundance of that phylum within the treatment group. Distinct visual patterns are present between treatment groups. Media abbreviations - LB: 25% Luria Broth; TSA: 10% Tryptic Soy; PSA: Psuedomonad Semi-selective; RM: Rhizosphere.

| ¼ Luria Broth | 1/10 Tryptic Soy Agar | Pseudomonad Selective Agar | Rhizosphere Media |
| --- | --- | --- | --- |
| 2.5g Tryptone  0.8g Yeast Extract  2.5g NaCl  15g Agar  10ug/mL Cycloheximide | 1.7g Tryptone  0.3g Soy Flour  0.5g NaCl  0.25g Dipotassium Phosphate  0.25g Glucose  15g Agar  10ug/mL Cycloheximide | 20g Peptone  1.4g MgCl  10g KSO4  10mL Glycerol  13g Agar  40ug/mL Ampicillin  12.5 ug/mL Chloramphenicol  10ug/mL Cycloheximide | 50g Arabidopsis Rhizosphere Soil (blended for homogeneity)  0.5g Dipotassium Phosphate  1g glucose  15g agar  10ug/mL Cycloheximide |

Table S1. Recipes for Solid Media

| **A) Cross-study Control** | **Flowering Time** | **Mean Individual Dry Weight** |
| --- | --- | --- |
| Whole Microbiome Control | 33.17+0.63 days | 0.0124+0.0022g |
| Cultivation Phase Control | 32.39+0.43 days | 0.0117+0.0009g |
| Revival Phase Control | 30.48+0.35 days | 0.0083+0.0013g |
| **B) PBS Control** |  |  |
| Cultivation PBS Control | 30.00+0.51 days | 0.0129+0.0038g |
| Revival PBS Control | 30.27+0.51 days | 0.0120+0.0036g |
| **C) Revival Phase** |  |  |
| LB-Plate Method | 31.38+0.37 days | 0.0081+0.0006g |
| LB-Liquid Method | 30.83+0.34 days | 0.0074+0.0005g |
| TSA-Plate Method | 30.63+0.37 days | 0.0073+0.0006g |
| TSA-Liquid Method | 30.40+0.34 days | 0.0065+0.0004g |
| PSA-Plate Method | 30.78+0.30 days | 0.0132+0.0027g |
| PSA-Liquid Method | 30.28+0.38 days | 0.0066+0.0004g |
| RM-Plate Method | - | - |
| RM-Liquid Method | - | - |

Table S2. Non-significant supplemental data


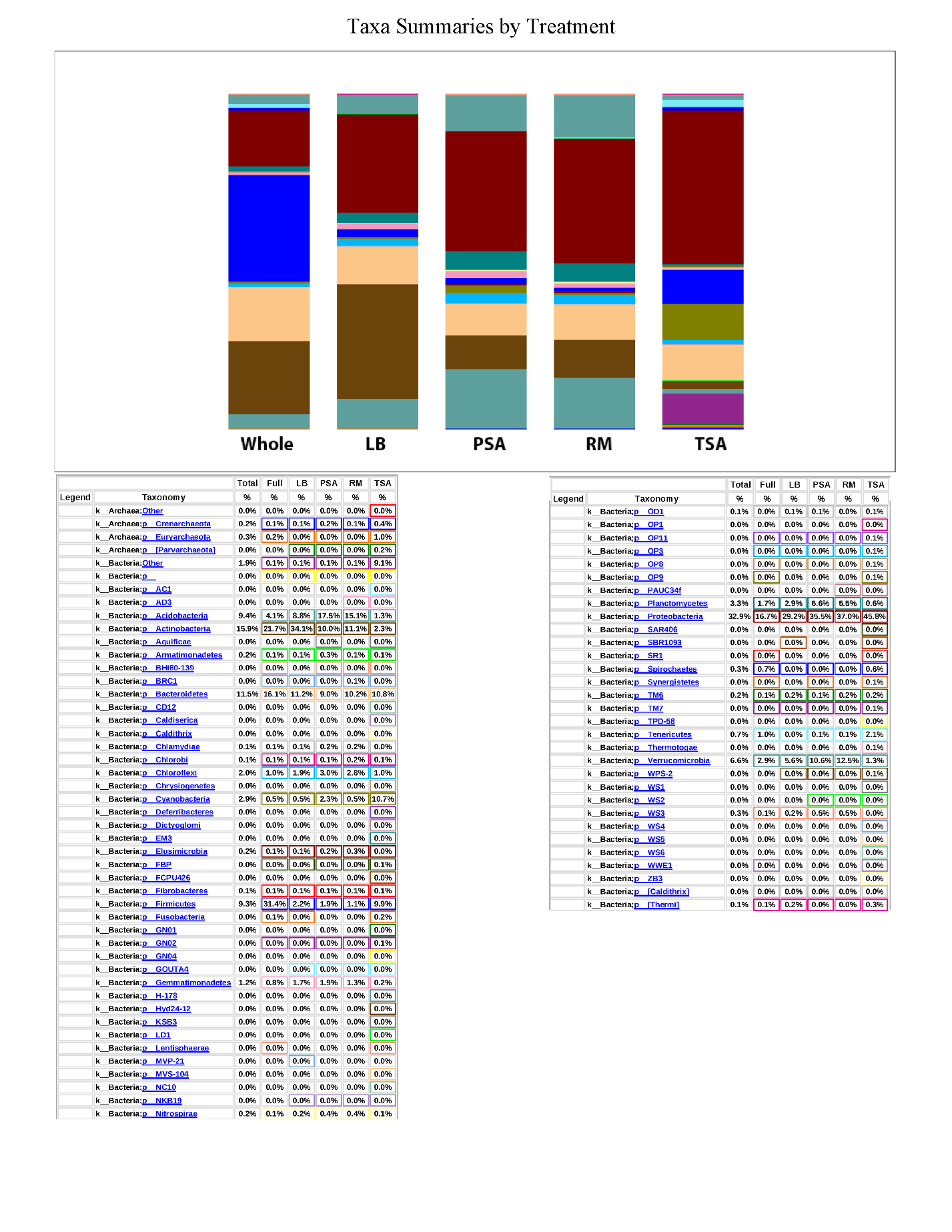

Supplement: Supplementary file 1 — (DOCX 676 kb) [file 248_2016_846_MOESM1_ESM.docx]
